# Right-Sided Aortic Arch Presenting as Chronic Cough in a 50-Year-Old Patient With DiGeorge Syndrome: A Report of a Rare Case

**DOI:** 10.7759/cureus.103028

**Published:** 2026-02-05

**Authors:** John Wahidy, Sawyer Longley, Muhammad Zohaib Anwar, Adrian DiVittorio

**Affiliations:** 1 Research, Alabama College of Osteopathic Medicine, Dothan, USA; 2 Medicine, Alabama College of Osteopathic Medicine, Dothan, USA; 3 Internal Medicine, Aga Khan University Medical College, Karachi, PAK; 4 Pulmonology and Critical Care, Mobile Infirmary, Mobile, USA

**Keywords:** chronic cough, congenital vascular anomaly, copd, digeorge syndrome, right-sided aortic arch, tracheal compression

## Abstract

A right-sided aortic arch is a rare congenital vascular anomaly that can be seen in individuals with 22q11.2 microdeletion syndromes, such as DiGeorge syndrome. Although most patients are asymptomatic, airway or esophageal compression may occur in a small subset of individuals. In this case, a 50-year-old male patient with DiGeorge syndrome presented with a persistent dry cough for roughly six weeks that was not improving despite medical treatment. Computed tomography (CT) imaging showed a right-sided aortic arch, and over several years, it progressively continued to compress the patient's trachea, which was confirmed via bronchoscopy. This case emphasizes the importance of considering anatomical anomalies in patients with unexplained chronic cough and emphasizes the necessity for collaboration among medical specialties to evaluate and treat this rare presentation.

## Introduction

DiGeorge syndrome is caused by a chromosome 22q11.2 deletion that occurs in around 1:4,000 births [1], which has a complex presentation with thymic aplasia, cardiac anomalies, facial abnormalities, and mild to moderate immune deficiency, and may include hypoparathyroidism. Cardiac anomalies occur in approximately 77% of patients with DiGeorge syndrome [1] and can present as tetralogy of Fallot, ventricular septal defects, interrupted aortic arch, truncus arteriosus, and vascular rings. These abnormalities can be accompanied by additional anomalies of the outflow structures, making treatment difficult, such as a right-sided aortic arch [1].

Embryologically, a right-sided aortic arch results from abnormal persistence of the right fourth aortic arch with regression of the left fourth aortic arch [2]. In individuals with normal anatomy, the aortic arch generally courses to the left of the trachea and esophagus, arching over the left main bronchus and anterior to the vertebral column [3].

Right-sided aortic arches are often clinically silent in patients with these abnormalities, but they can occasionally manifest as cough, dyspnea, and even chest pain. One study conducted in 2012 at a hospital in Turkey found that right-sided aortic arches were symptomatic in 13 patients presenting to the hospital, and “cough” was the chief complaint in six patients over a four-year period [3].

Right-sided aortic arches are considered to be very rare, as they are seen in 0.01%-0.1% of the population [2,4,5]. That number is dramatically higher in individuals who have a 22q11.2 deletion. In a study of 37 individuals with a right-sided aortic arch, 11 participants had the chromosomal deletion [6]. Symptomatic cases of a right aortic arch are uncommon in adults, making clinical findings all the more important. Chronic cough is a possible symptom and may be due to esophageal compression. In this case report, we present a patient with a chronic cough due to a right-sided aortic arch.

## Case presentation

The patient was a 50-year-old man with DiGeorge syndrome, obstructive sleep apnea (OSA) on continuous positive airway pressure (CPAP), and a 30-year pack smoking history who presented to the pulmonology clinic for a chief complaint of cough. The cough had been ongoing for approximately 1.5 months. The cough was mainly dry in nature and nonproductive, but occasionally produced some phlegm. He reported some symptoms of breathlessness. He underwent testing for COVID, influenza, and strep throat, all of which came back negative. He had been seen by a local otolaryngologist and underwent laryngoscopy, which was reported to be unremarkable. He was started on inhaled controller therapy and a short-acting bronchodilator as needed. Initial differential diagnoses included asthma, chronic obstructive pulmonary disease, and gastroesophageal reflux disease based on his clinical presentation and smoking history. A follow-up visit was scheduled in eight weeks to evaluate if there had been any improvement in his cough. In between the visits to the pulmonary clinic, he had visited his primary care physician, who started him on doxycycline hyclate 100 mg tablets twice a day for 10 days due to some bronchitis symptoms of recurrent hoarseness and sinus congestion. Despite treatment with inhaler therapy, antibiotics, and proton pump inhibitors, his persistent cough remained.

**Figure 1:**
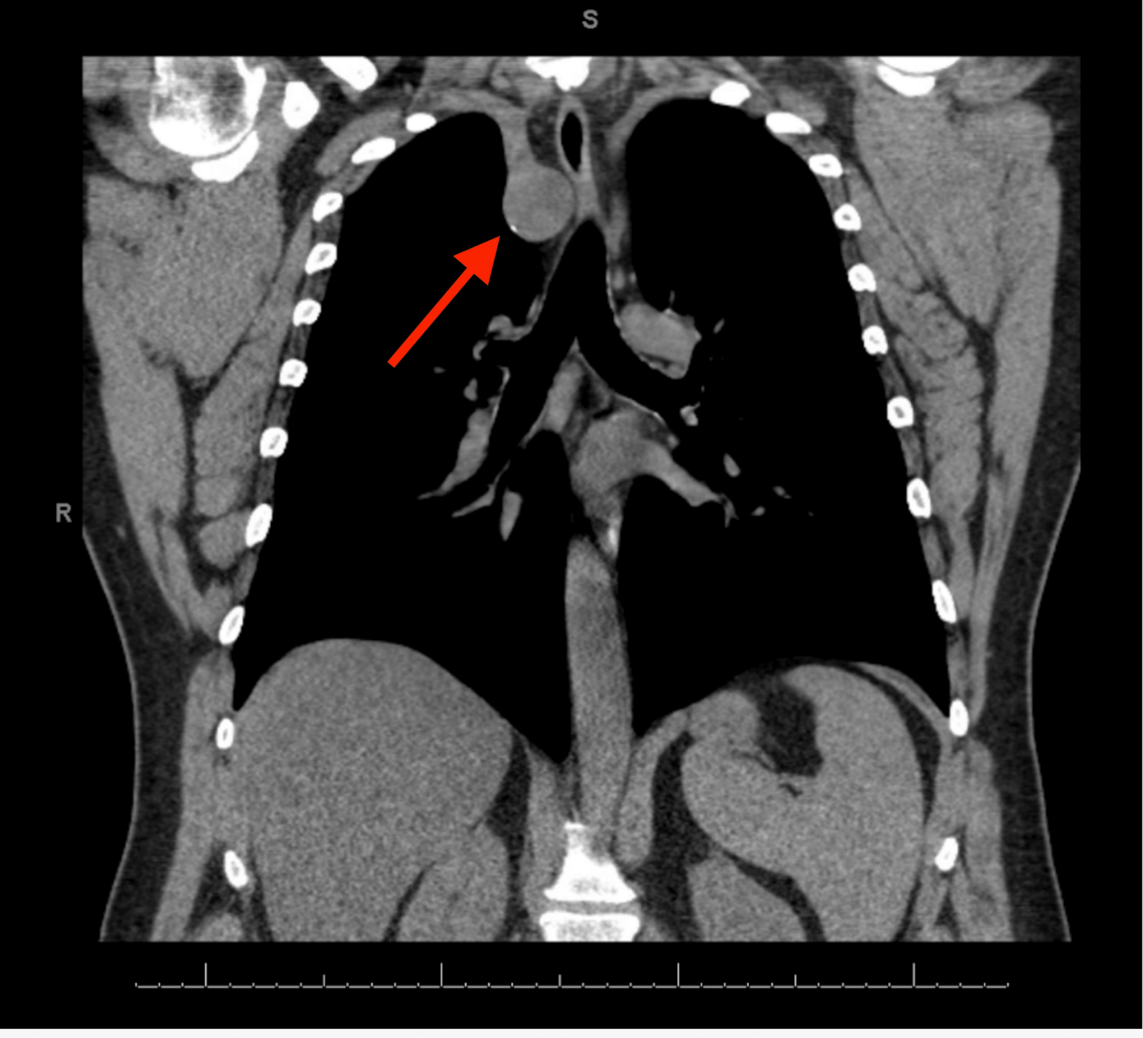
Coronal CT image from 2025 demonstrating a right-sided aortic arch (red arrow) with significant tracheal compression. CT: computed tomography

**Figure 2:**
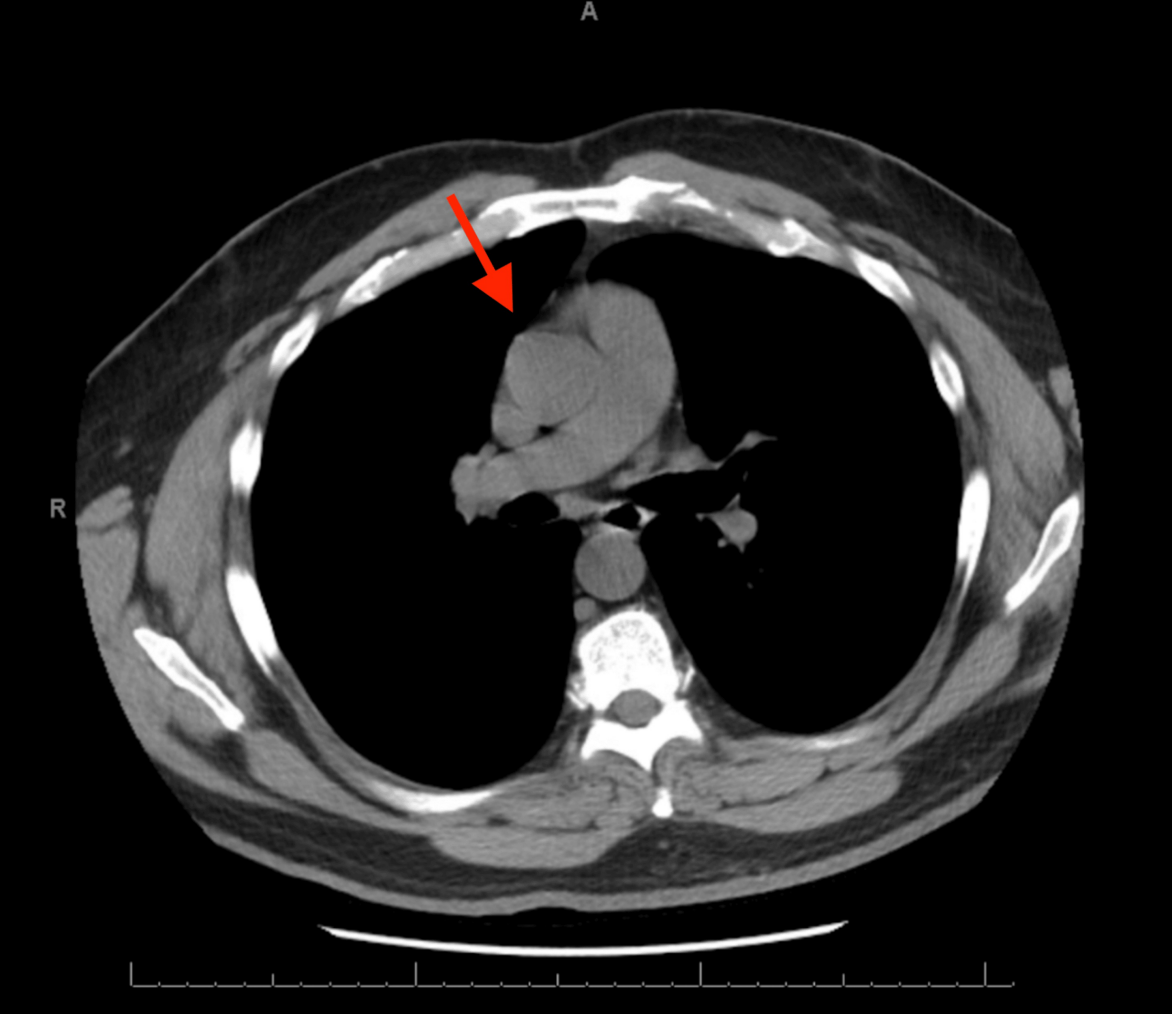
Axial CT image from 2019 demonstrating an incidental right-sided aortic arch (red arrow) without significant tracheal compression. CT: computed tomography

At his follow-up visit, he still had a persistent, dry cough that was refractory to treatment with inhalers and antibiotics. A computed tomography (CT) scan of the chest that had been ordered a month before his second visit showed a right-sided aortic arch with significant compression of his upper cervical trachea (Figure 1). Given the significant tracheal compression, we hypothesized that it could be causing his significant cough. After reviewing his chart, he had been seen in a clinic in 2022 for globus sensation and underwent a neck CT that showed no significant findings. He had also undergone a CT scan of the chest in 2019 at the Emergency Department after falling through a drywall ceiling and developing chest pain. The CT then showed an incidental finding of a right aortic arch with slight tracheal compression (Figure 2). From the older CT to the newer CT, he had gained around 48 lbs, which could be contributing to the change in the arch, or it could just be a normal development of the arch.

**Figure 3:**
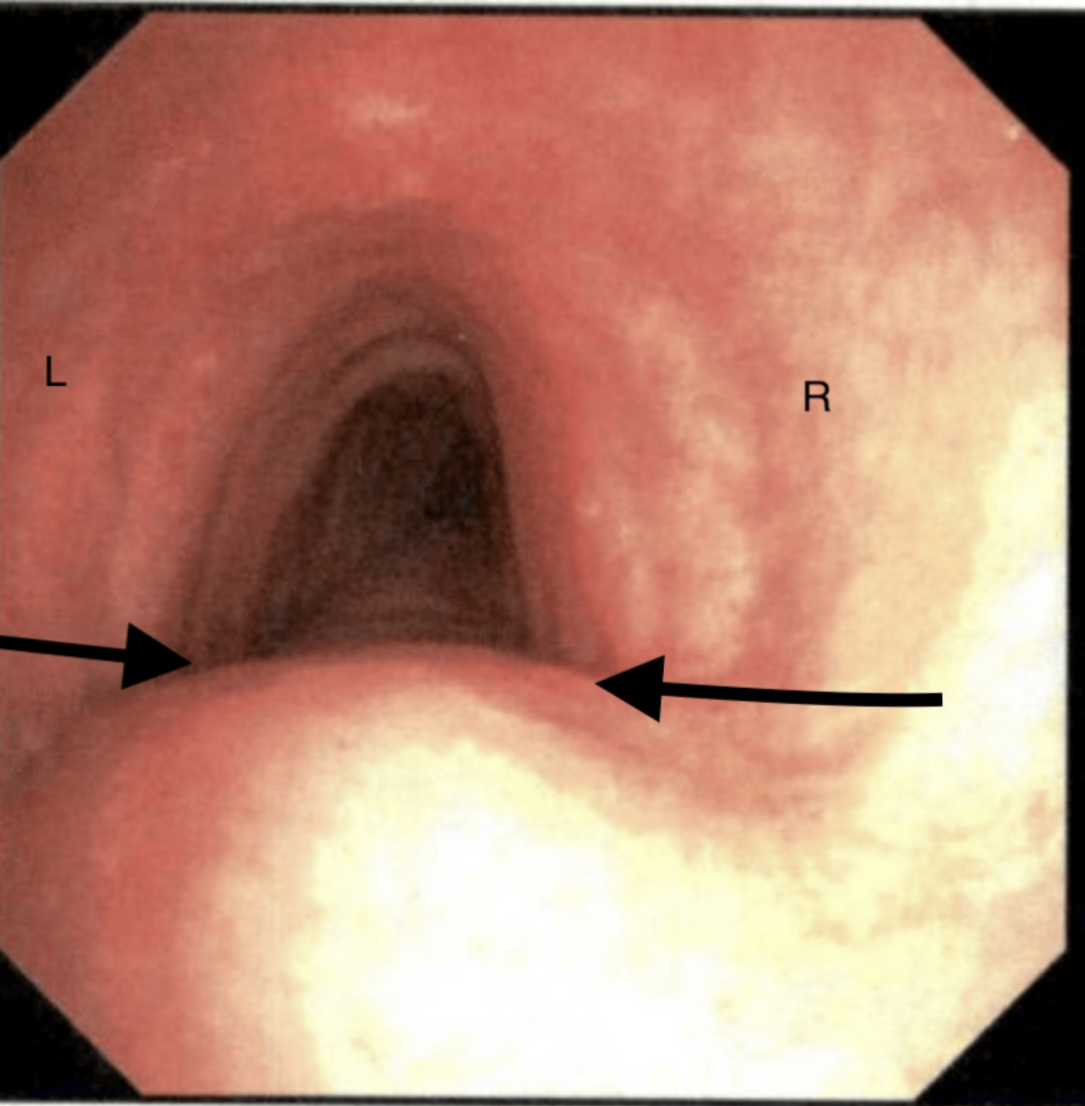
Bronchoscopy from 2025 demonstrating tracheal narrowing due to extrinsic compression (black arrows). The left (L) and right (R) sides of the airway are labeled for anatomical orientation.

After discussing options with the patient, it was decided to perform a bronchoscopy to determine whether any intrapulmonary causes were eliciting his cough. Bronchoscopy performed on a three-month follow-up visit confirmed dynamic narrowing of the trachea without it collapsing while coughing. There was the right external compression of the trachea as well (Figure 3). Mucosal cobblestoning was also seen, suggesting chronic airway irritation, but these findings alone do not account for the severe symptoms. Cardiothoracic surgery was consulted and agreed to evaluate him for potential surgery and translocation of his right-sided aortic arch. Exercise, weight loss, and dietary changes can also improve patients' symptoms and decrease tracheal dysfunction (Table 1).

**Table 1:** Timeline of events. CT: computed tomography; GERD: gastroesophageal reflux disease

Timeline	Findings
Remote history	CT of chest shows incidental right-sided aortic arch (Figure *2*)
Remote history	Seen for globus sensation, CT showed no abnormalities
Presentation day	Seen for a persistent dry cough and started on budesonide/glycopyrrolate/formoterol fumarate
Follow-up month 1	Started on doxycycline hyclate/omeprazole for possible bronchitis/GERD; however, symptoms remained
Follow-up month 2	CT of chest showing right-sided aortic arch with significant compression (Figure *1*)
Follow-up month 3	Bronchoscopy confirmed narrowing and compression of the trachea (Figure *3*)
Ongoing	Cardiothoracic surgery consult and potential surgery/translocation

## Discussion

Most cases of a right-sided aortic arch are asymptomatic and do not require treatment [3,7]. Although uncommon, symptomatic presentation in adulthood is usually related to progressive compression of the airway or esophagus. The majority of published reports describe pediatric presentations, such as this four-year-old patient with a right-sided aorta who underwent division of an atretic anterior aortic arch and posterior aortopexy at 10 months of age. It was then decided to translocate the aortic arch anterior to the trachea and esophagus. The patient had some reduced left vocal cord movement post-surgery but was able to be discharged home in good clinical condition [8]. Due to the patient’s significant cough and tracheal compression on CT scan, a procedure to mitigate symptoms by translocating the right-sided aorta, similar to the one above, may be warranted to improve the patient’s quality of life. In patients with normal anatomical (left-sided) aortic arches, it courses to the left of the trachea and esophagus, arching over the left main bronchus and anterior vertebral column [2].

In contrast, adult-onset symptoms are not reported as frequently and can be attributed to the mass effect of adjacent anatomical structures or even gradual anatomical changes as one ages. For this present case, serial CT imaging showed evidence of progressive tracheal compression over several years, which can be attributed to the right-sided aortic arch as the degree of vascular compression increased. This slow progression likely explained the delayed onset of symptoms despite the anomaly being congenital.

When deciding on how to treat an individual with aortic arch anomalies, those who experience progressive symptoms such as dyspnea or chronic cough show no signs of improvement on inhaled corticosteroids and long-acting beta agonists [3]. Further evaluation with imaging can demonstrate esophageal compression, and surgery should be considered to improve the patient's condition [9,10]. One finding reports of a 25-year-old male patient who was experiencing dysphagia, and a CT angiography (CTA) revealed a right-sided aortic arch with a left subclavian artery moving posteriorly to the esophagus. This patient quickly had a left subclavian bypass done, and his symptoms had resolved [11]. These reports are critical when diagnosing a right-sided aortic arch in order to treat all who have this unique anomaly. The present case is notable for adult-onset respiratory symptoms with objective demonstration of progressive tracheal compression on serial imaging. This delayed presentation highlights that previously incidental congenital vascular anomalies can eventually become relevant later in life and be considered when evaluating chronic coughs in adults that do not improve with treatment.

## Conclusions

This case illustrates the diagnostic challenge posed by a persistent cough in an adult with DiGeorge syndrome and a right-sided aortic arch. Although right-sided aortic arches are usually asymptomatic, our patient’s imaging showed notable tracheal compression. The persistence of cough despite optimized medical therapy suggests a structural contribution from the anomalous arch. In such cases, conservative measures may offer some limited relief, and surgical intervention should also be considered to alleviate mechanical compression. This case adds to the limited literature describing adult-onset symptoms related to right-sided aortic arches and emphasizes the importance of a thorough multimodal evaluation in guiding management. This patient's presentation highlights the importance of considering structural causes of chronic cough, emphasizing how repeat CT scans can track progressive vascular compression. Continued interdisciplinary collaboration across fields such as cardiology, pulmonary, and vascular systems is highlighted by this case, as it demonstrates the value of coordination and treatment for patients with symptomatic vascular anomalies.
